# Outcomes and Laboratory and Clinical Findings of Asthma and Allergic Patients Admitted With Covid-19 in a Spanish University Hospital

**DOI:** 10.3389/fphar.2020.570721

**Published:** 2020-09-16

**Authors:** Jesús Miguel García-Menaya, Concepción Cordobés-Durán, Juan Francisco Rangel-Mayoral, Elena García-Martín, José A. G. Agúndez

**Affiliations:** ^1^ Allergy Service, Badajoz University Hospital, ARADyAL Instituto de Salud Carlos III, Badajoz, Spain; ^2^ Allergy Service, Hospital de Don Benito, ARADyAL Instituto de Salud Carlos III, Don Benito, Spain; ^3^ Hospital Pharmacy Service, Badajoz University Hospital, Badajoz, Spain; ^4^ University Institute of Molecular Pathology Biomarkers, UNEx, ARADyAL Instituto de Salud Carlos III, Cáceres, Spain

**Keywords:** coronavirus disease 2019, severe acute respiratory syndrome coronavirus 2, asthma, allergy, clinical outcome

## Abstract

Individual susceptibility and clinical outcome of Covid-19 are variable and mortality is also very variable across countries, being particularly high in Spain. Comorbidities might increase the risk for less favourable outcomes, but it has been reported that patients with antecedents of asthma or allergic diseases were under-represented among hospitalized Covid-19 patients. Aiming to compare the clinical evolution of patients with antecedents of asthma or allergic diseases and patients without these antecedents, we analyzed a series of 113 consecutive patients with Covid-19 in a regional hospital in Spain. We collected and analyzed the putative effect of the 16 most common co-morbidities, previous treatment with 33 drug classes, symptoms, radiological, and laboratory findings at admission and drug therapy after admission. Predictors of long hospital stays were older age (P = 0.002), low oxygen saturation (P = 0.001) and bilateral radiological findings at admission (P = 0.023). Predictors of Intensive Care Unit (ICU) admission were the previous use of calcium-channel blockers (P = 0.005), proton pump inhibitors (P = 0.017), low oxygen saturation (P = 0.002), high leukocyte count (P = 0.011), and high D-dimer values (P = 0.005). Predictors of mortality were older age (P = 0.001), antecedents of cerebrovascular disorders (P = 0.034), previous use of oral anticoagulants (P = 0.009) or selective serotonin reuptake inhibitors (P = 0.003), and increased levels of interleukin-6 (P = 0.001). Patients with antecedents of allergic diseases were about ten years younger (P = 0.003) and had fewer comorbidities (P = 0.026) than the rest of the patients. In conclusion, antecedents of allergic diseases might influence hospitalization risk in relatively young patients.

## Introduction

At the beginning of December 2019, a series of pneumonia cases of unknown origin were found in Wuhan, Hubei, China. Patient clinical presentations were similar to viral pneumonia. About three weeks later, by January 2, 2020, at least 41 patients had been admitted to hospital with similar clinical findings (Huang et al., 2020). Subsequently, they were confirmed to have an infection caused by a novel coronavirus, identified on January 7 by the Chinese Center for Disease Control and Prevention, and initially named 2019-nCoV (Chen et al., 2020). This new pathogen was identified as an enveloped RNA betacoronavirus (Lu et al., 2020). This novel coronavirus is a virus with phylogenetic similarity to SARS-CoV, and was isolated in human airway epithelial cells. It is the seventh member of the family of coronavirus with the ability to infect humans (Zhu et al., 2020). By February 11 the World Health Organization (WHO) named it severe acute respiratory syndrome coronavirus 2 (SARS-CoV-2) and the disease caused coronavirus disease 2019 (Covid-19) (^1^).

Previous epidemics by another two betacoronaviruses SARS-CoV and MERS-CoV affected more than 10,000 patients (Drosten et al., 2003; Zaki et al., 2012), with high rates of mortality (about 10% for SARS-CoV and 37% for MERS-CoV, respectively). The origin of this novel virus SARS-CoV-2 is still unclear. It shares an 87.99% identity genetic sequence with the bat SARS coronavirus (Tan et al., 2020), although at this moment it is not known whether other animals have acted as possible vectors.

By January 31, the first patient with Covid-19 was diagnosed in Spain (Spiteri et al., 2020). Since then, Spain, like other southern European countries, has had a high rate of prevalence and mortality by Covid-19 (Ceylan, 2020; Sotgiu et al., 2020). At the time of writing this manuscript (June 7^th^) 241,550 patients have been diagnosed with Covid-19 by reverse transcription-polymerase chain reaction (RT-PCR) and 27,136 have died due to Covid-19 (^2^).

Different studies have been published describing clinical and analytic characteristics of Covid-19 patients mainly in China (Chen et al., 2020; Guan et al., 2020b; Huang et al., 2020; Wang et al., 2020a; Wang et al., 2020b; Zhao et al., 2020), and also Western countries (Goyal et al., 2020; Lechien et al., 2020; Richardson et al., 2020). Description of geographical differences in the clinical presentation and outcome is crucial, as it seems that this virus has a high rate of mutation and that the virus seems to have a different clinical impact in different countries and in subjects with different ethnicities [Niedzwiedz et al., 2020; (^3^); Moorthy et al., 2020].

Initial studies carried out in China have assessed the asthmatic and allergic antecedents of admitted patients (Li et al., 2020; Zhang et al., 2020). Surprisingly, these two studies report very few asthmatic or allergic patients in Wuhan. Zhang et al. reported comorbidities of 140 hospitalized Covid-19 patients in Wuhan and urticaria and drug hypersensitivity were only self-reported by 1.4 and 11.4% of patients, respectively. No patient reported asthma or any other allergic disease (Zhang et al., 2020). Li et al. have described 548 Covid-19 patients admitted to the hospital. The prevalence of asthma in those patients was only 0.9%, which is much lower than the asthma prevalence of Wuhan, which is more than 6% (Li et al., 2020).

Such initial evidence has led some authors to wonder whether chronic respiratory diseases (included asthma) or their treatments affect the risk of SARS-CoV-2 infection or its evolution (Halpin et al., 2020a). An initial experimental study was carried out with the hypothesis that one possible explanation for the low prevalence of asthma and other allergic diseases in Covid-19 patients could be a reduced angiotensin-converting enzyme-2 (ACE2) gene expression in the airway cells of these patients (Jackson et al., 2020), but another experimental study has found opposite results (Sajuthi et al., 2020). Nevertheless, some published articles indicate that asthma increases the risk or the severity of Covid-19 (Pennington, 2020), or suggest that asthma is a risk factor for severe outcomes (Hegde, 2020; Johnston, 2020), although the three articles cited do not provide specific data supporting this hypothesis. In this respect, a systematic review has been published on inhaled corticosteroids (ICS) and Covid-19, concluding that at present there is no evidence that previous or continued use of ICS is beneficial or detrimental to SARS-CoV-2 infection (Halpin et al., 2020b).

As far as we are aware, no study has specifically assessed whether hospitalized Covid-19 patients with asthma or allergic disorders present different laboratory or clinical findings, as compared to patients without antecedents of asthma or allergic disorders. In this study, we analyzed the frequency, symptoms, laboratory parameters, and radiological findings at hospital admission, and the influence of comorbidities, drug therapy before and after hospital admission, in the clinical evolution of patients stratified according to the presence of antecedents of asthma or allergic disorders.

The main aim of this study was to compare the characteristics, clinical presentation and outcome of Covid-19 patients with allergic disorders, patients with no allergic antecedents and overall patients. Also, we analyzed the putative effect of comorbidities, drug therapy before admission, symptoms, laboratory and radiological findings, drug therapy used for Covid-19 on clinical outcomes. We evaluated as main outcomes, the time until hospital discharge, ICU admission, and mortality.

## Methods

### Subjects

The study was conducted at Badajoz University Hospital Complex, which is the largest hospital in Extremadura, Spain. This hospital covers an area with 271,885 inhabitants in Badajoz. All consecutive patients admitted to the Badajoz University Hospital Complex from March 16 to April 24, and diagnosed with Covid-19 by RT-PCR and/or serological tests were assessed. We excluded from the analyses patients who remained in the hospital when this manuscript was written because the clinical outcome was still uncertain. We also excluded patients directly discharged from the emergency department with no hospital admittance.

The final cohort included 113 patients. One hundred and eleven patients were diagnosed with Covid-19 by RT-PCR taken from pharyngeal and/or nasal swab specimens and only 2 patients were diagnosed exclusively by clinical and laboratory findings in addition to serological positive test. Diagnostic tests were performed in the microbiology laboratory of Badajoz University Hospital. In the case of initial negative RT-PCR, the tests were repeated during hospitalization if there was a high clinical probability or if the initial test was considered to be a false negative. The Research Ethics Committee of the Badajoz Health Area approved this study.

### Data Collection

We recorded information on the date of hospital admission, hospital discharge due to clinical improvement or death, demographic data such as age and sex, laboratory findings, radiological findings (chest X-ray and/or computed tomographic scan), drug therapy before admission including 33 drug groups, treatment received for COVID-19 management, and previous comorbidities including 16 types of antecedents plus allergic disorders (including asthma, allergic rhinitis, food allergy, urticarial, and drug allergy), and signs and symptoms at admission (**Table 1**). A single researcher with personal clinical experience in the management and treatment of Covid-19 patients collected all this information, which was obtained by homogeneous reviewing from patients’ electronic clinical histories, including medical reports, clinical and treatment notes, and laboratory findings (14 different variables) recorded in a Healthcare Informatics System called JARA. Intensive Care Unit (ICU) admissions were also recorded. Laboratory findings correspond to analyses carried out on admission (within first 24 h of admission) or the first time that they had been performed (number of leukocytes, lymphocytes, eosinophils and platelets, alanine aminotransferase and aspartate aminotransferase, lactate dehydrogenase (LDH), creatine kinase (CK), C-reactive protein (CRP), serum creatinine, D-dimer, ferritin, procalcitonin (PCT) and interleukin-6 (Il-6). The clinical laboratory of Badajoz University Hospital performed all these analyses.

**Table 1:** Age, gender and comorbidities of the patients included in this study.

	Overall patients(n = 113)	Patients with allergic disorders (n = 24)	Other patients(n = 89)	Intergroup comparison values
Age (mean ± SD); range	67.62 ± 16.05; 28-99	59.21 ± 16.21; 28-86	69.89 ± 15.32; 29-99	P = 0.003^1^
Gender (women/men)	56/57	14/10	42/47	P = 0.333^2^
Comorbidity excluding asthma or allergy, (yes/no)	93/20	16/8	77/12	P = 0.024^2^
Antecedents of hypertension, (yes/no)	61/52	8/16	53/36	P = 0.022^2^
Antecedents of heart diseases, (yes/no)	28/85	7/17	21/68	P = 0.575^2^
Antecedents of dyslipidemia, (yes/no)	27/86	2/22	25/64	P = 0.044^2^
Antecedents of type II diabetes, (yes/no)	25/88	2/22	23/66	P = 0.067^2^
Antecedents of renal diseases, (yes/no)	20/93	0/24	20/69	P = 0.010^2^
Antecedents of prothrombotic disorders, (yes/no)	18/95	7/17	11/78	P = 0.046^2^
Antecedents of respiratory diseases^3^, (yes/no)	15/98	3/21	12/77	P = 0.900^2^
Antecedents of cerebrovascular diseases, (yes/no)	13/100	2/22	11/78	P = 0.583^2^
Antecedents of cognitive impairment, (yes/no)	13/100	2/22	11/78	P = 0.583^2^
Antecedents of psychiatric diseases, (yes/no)	11/102	2/22	9/80	P = 0.794^2^
Antecedents of hormonal disorders, (yes/no)	11/102	1/23	10/79	P = 0.300^2^
Antecedents of neurological diseases, (yes/no)	10/103	2/22	8/81	P = 0.920^2^
Antecedents of digestive diseases, (yes/no)	10/103	1/23	9/80	P = 0.363^2^
Antecedents of neoplastic diseases, (yes/no)	10/103	0/24	10/79	P = 0.085^2^
Antecedents of morbid obesity, (yes/no)	8/105	2/22	6/83	P = 0.787^2^
Antecedents of anaemia, (yes/no)	7/106	1/23	6/83	P = 0.642^2^

^1^T-test for independent samples.

^2^Pearson’s Chi-square test.

^3^Patients with asthma were excluded from this group.

### Statistical Analysis

Analyses were performed using the Statistical Package for the Social Sciences (SPSS) version 1.0.0.1327 for Windows (SPSS Inc., Chicago, IL, USA). Comparisons were considered statistically significant when P-values were lower than 0.05. Intergroup comparisons were carried out by using the T-test for unpaired samples or the Chi-square test when appropriate. Associations between variables were assessed by using linear or logistic regression for continuous or categorical variables, respectively. Because some factors are likely to be related (for instance antecedents of cardiovascular disorders and the use of statins, platelet antiaggregants, beta-blockers and/or calcium channel blockers, we performed multivariate analyses.

## Results

We identified 24 patients with antecedents of allergic disorders in the study group. This represents roughly 21% of patients, which is consistent with the frequency of about 21.6% of individuals with antecedents of allergic disorders in Spain (Gaig et al., 2004). Of these patients, 11 had a drug allergy (four patients with allergy to beta-lactams, two patients with allergy to NSAID, and five patients with allergy to other drugs and contrast media). Five patients had antecedents of rhinitis and asthma, three patients rhinitis and two patients asthma, two patients had a seasonal allergy and two patients had urticaria. One patient presented asthma plus rhinitis plus urticaria. The specific pharmacological treatment received by these patients is summarized in **Table 2**.

**Table 2:** Drug therapy before hospital admission for all participants in this study.

	Overall patients(n = 113)	Patients with allergic disorders (n = 24)	Other patients(n = 89)	Intergroup comparison values
Drugs used in allergic diseases				
Inhaled corticosteroids (yes/no)	16/97	8/16	8/81	P = 0.002
Inhaled Beta-2 agonists (yes/no)	15/98	9/15	6/83	P = 0.001
Inhaled Anticholinergics (yes/no)	9/104	4/20	5/84	P = 0.076
Antihistamine drugs (yes/no)	4/109	2/22	2/87	P = 0.152
Anti-leukotrienes agents (yes/no)	1/112	1/23	0/89	P = 0.053
Omalizumab (yes/no)	1/112	1/23	0/89	P = 0.053
Drugs used in cardiovascular diseases				
Statins (yes/no)	39/74	8/16	31/58	P = 0.891
Angiotensin-II-receptor antagonists (sartans) (yes/no)	37/76	5/19	32/57	P = 0.161
Diuretics (yes/no)	29/84	2/22	27/62	P = 0.029
Platelet Antiaggregants (yes/no)	27/86	5/19	22/67	P = 0.692
Beta-blockers	24/89	6/18	18/71	P = 0.612
Calcium channel blockers (yes/no)	18/95	2/22	16/73	P = 0.252
Oral anticoagulants (yes/no)	14/99	6/18	8/81	P = 0.035
Angiotensin-converting enzyme (ACE) inhibitors (yes/no)	12/101	2/22	10/79	P = 0.682
Other vasodilators (yes/no)	9/104	1/23	9/80	P = 0.363
Pain relief medications				
Non-steroidal anti-inflammatory drugs (yes/no)	35/78	6/18	29/60	P = 0.476
Opiates (yes/no)	16/97	5/19	11/78	P = 0.291
Psychiatric medications				
Benzodiazepines (yes/no)	38/75	6/18	32/57	P = 0.313
Selective serotonin reuptake inhibitors (SSRI)s (yes/no)	23/90	5/19	18/71	P = 0.948
Antipsychotic agents (yes/no)	10/103	2/22	8/81	P = 0.920
Other antidepressants (not SSRI)s	7/106	2/22	5/84	P = 0.624
Drug used in neurological diseases				
Anti-Parkinson Drugs (yes/no)	6/107	0/24	6/83	P = 0.191
Antiepileptic drugs (yes/no)	5/108	1/23	4/85	P = 0.945
Other drugs				
Proton pump inhibitors (yes/no)	54/59	7/17	47/42	P = 0.040
Oral antihyperglycemic drugs (yes/no)	25/88	3/21	22/67	P = 0.201
Antibiotics (yes/no)	11/102	1/23	10/79	P = 0.300
Levothyroxine (yes/no)	9/104	0/24	9/80	P = 0.104
Antiandrogens (yes/no)	9/104	0/24	9/80	P = 0.104
Anticholinergics (yes/no)	7/106	0/24	7/82	P = 0.156
Adrenergic alpha 1-antagonists (yes/no)	5/108	0/24	5/84	P = 0.235
Immunosuppressants (yes/no)	5/108	1/23	4/85	P = 0.945
Oral corticosteroids (yes/no)	3/110	0/24	3/86	P = 0.362

**Table 1** shows age, gender, and comorbidities of overall patients, patients with allergic disorders, and the rest of the patients. Intergroup comparison between the patients with allergic disorders and the rest of the patients revealed statistically significant differences regarding age at onset, patients with allergic disorders being roughly ten years younger. The presence of other comorbidities was significantly less frequent in patients with allergic disorders, with an Odds Ratio (OR, 95% C.I.) equal to 0.32 (0.11–0.90). Sixteen different comorbidities were observed in this study (**Table 1**), and four of these were present with statistically different frequencies between the patients with allergic disorders and the rest of the patients. These were antecedents of hypertension, dyslipidemia, renal diseases, and prothrombotic disorders. The statistical significance of these differences was small, and could be attributed to the very significant difference in age between the subgroups of patients. Overall, these findings suggest that patients with antecedents of allergic disorders constitute a distinct group composed of younger and healthier patients, as compared to the general group of patients. This suggests that antecedents of allergic disorders could be predisposing factors for hospitalization related to COVID-19.

With the aim of evaluating the potential impact of previous drug therapy in the clinical course of COVID-19 disease, we collected information on the use of any drugs received by participants before hospital admission. This information, comprising 33 drug classes, is summarized in **Table 2**. Regarding drugs usually used in allergic diseases, no statistically significant differences were observed between both subgroups of patients except for inhaled corticosteroids and inhaled beta-2 antagonists. For the rest of the drug classes, we observed weakly significant differences in the use of diuretics and proton pump inhibitors (less frequent in patients with antecedents of allergy) and oral anticoagulants, which were more frequently used in patients with antecedents of allergy.

**Table 3** shows the clinical symptoms of patient subgroups at hospital admission. Thirteen symptoms were observed and there were no statistically significant differences between subgroups, thus suggesting that patients with allergic disorders present with the same symptoms as the rest of patients.

**Table 3:** Symptoms of the patients included in this study when admitted to the Hospital.

	Overall patients(n = 113)	Patients with allergic disorders (n = 24)	Other patients(n = 89)	Intergroup comparison values
Fever (yes/no)	81/32	18/6	63/26	P = 0.684^1^
Dry cough (yes/no)	77/36	17/7	60/29	P = 0.750^1^
Dyspnea (yes/no)	68/45	15/9	53/26	P = 0.793^1^
Overall discomfort	35/78	6/18	29/60	P = 0.476^1^
Asthenia (yes/no)	20/93	1/23	19/70	P = 0.050^1^
Diarrhoea (yes/no)	20/93	6/18	14/75	P = 0.291^1^
Musculoskeletal pain	18/95	5/19	13/76	P = 0.459^1^
Anosmia	10/103	1/23	9/80	P = 0.363^1^
Ageusia	10/103	1/23	9/80	P = 0.363^1^
Headache	5/108	1/23	4/85	P = 0.945^1^
Nauseas and/or vomits	4/109	2/22	2/87	P = 0.152^1^
Odynophagia	4/109	2/22	2/87	P = 0.152^1^
Exanthema	1/112	0/24	1/88	P = 0.602^1^

^1^ Pearson’s Chi-square test.

**Table 4** shows the laboratory and radiological findings of the patient subgroups at hospital admission. No statistically differences were observed between these subgroups. **Table 5** shows the drugs received after hospital admission and, again, no statistically significant differences were observed.

**Table 4:** Laboratory and radiological findings of the patients included in this study when admitted to the Hospital or the first time that they had been performed.

	Overall patients(n = 113)	Patients with allergic disorders (n = 24)	Other patients(n = 89)	Intergroup comparison values
Temperature °C	37.10 (1.35)	37.35 (1.67)	37.10 (1.38)	P = 0.341^1^
Oxygen saturation (%)	94.0 (7.0)	94.0 (5.0)	93.0 (7.3)	P = 0.116^1^
Leukocytes per microliter	6890 (3890)	6790 (3838)	6890 (3840)	P = 0.818^1^
Lymphocytes per microliter	1,040 (650)	985 (845)	1,060 (570)	P = 0.659^1^
Eosinophils per microliter	10.0 (65.0)	10.0 (37.5)	10.0 (70)	P = 0.332^1^
Platelet count per microliter	205,000 (100,000)	199,000 (87,250)	207,000 (102,000)	P = 0.788^1^
Aspartate Aminotransferase, IU/L	32.0 (27.75)	31.00 (26.25)	33.00 (27.75)	P = 0.708^1^
Alanine Aminotransferase, IU/L	25.00 (21.50)	26.50 (22.25)	25.00 (19.00)	P = 0.924^1^
Lactate dehydrogenase, IU/L	294.0 (171.0)	271.0 (148.8)	311.0 (205.0)	P = 0.058^1^
Creatine Phosphokinase, IU/L	82.50 (133.75)	86.50 (135.75)	77.50 (134.25)	P = 0.793^1^
C-reactive protein, mg/L	71.40 (115.85)	54.95 (106.75)	78.00 (114.95)	P = 0.436^1^
Creatinine, mg/dL	1.01 (0.50)	0.86 (0.39)	1.02 (0.65)	P = 0.367^1^
D-dimer, ng/mL	903 (1,267.0)	651 (747.5)	1,077 (1,625.5)	P = 0.315^1^
Ferritin, ng/mL	518.0 (803.0)	568.0 (860.3)	475 (782.8)	P = 0.880^1^
Procalcitonin, ng/mL	0.12 (0.24)	0.08 (0.33)	0.12 (0.21)	P = 0.859^1^
Interleukin-6, pg/mL	24.36 (64.81)	24.12 (109.94)	24.36 (60.48)	P = 0.530^1^
No radiological findings	17/96	5/19	12/77	P = 0.371^2^
Unilateral radiological findings	27/86	3/21	24/65	P = 0.140^2^
Bilateral radiological findings	46/67	8/16	38/51	P = 0.407^2^

^1^T-test for independent samples.

^2^Pearson’s Chi-square test.

Laboratory data correspond to median (IQR).

**Table 5:** Drug therapy after admission.

	Overall patients(n = 113)	Patients with allergic disorders (n = 24)	Other patients(n = 89)	Intergroup comparison values
Hydroxychloroquine (yes/no)	100/13	21/3	79/10	P = 0.863^1^
Azithromycin (yes/no)	96/17	22/2	74/15	P = 0.300^1^
Lopinavir plus ritonavir (yes/no)	64/49	14/10	50/39	P = 0.850^1^
Corticosteroids (yes/no)	35/78	8/16	27/62	P = 0.778^1^
Interferon beta (yes/no)	7/106	1/23	6/83	P = 0.642^1^
Tocilizumab (yes/no)	5/108	0/24	5/84	P = 0.235^1^

^1^Pearson’s Chi-square test.

Regarding clinical outcomes, we evaluated the days of recovery until hospital discharge, admission to the ICU, and mortality. The importance of evaluating the days of recovery until hospital discharge and admission to the ICU lies in the fact that these are critical factors to determine the availability of health resources, and therefore to identify predictors of these parameters could be of great importance in health planning. Regarding mortality, 20 out of the 113 patients died, but no statistically significant differences between the death frequency for patients with allergic disorders and the rest of the patients were observed (**Table 6**). Overall, the mortality rate was strikingly high as compared to that reported in other studies (Chen et al., 2020; Goyal et al., 2020; Guan et al., 2020b; Huang et al., 2020), but it is consistent with the fact that only patients with severe symptoms were admitted to the hospital and that Spain has one of the highest mortality rates worldwide (Sotgiu et al., 2020). We then analyzed the putative impact of all covariates in these clinical outcome parameters, including comorbidities, drug therapy before admission, symptoms, laboratory and radiological findings at admission, and drug therapy after admission. All analyses were performed for the whole study group, the group of patients with antecedents of allergy, and the rest of the patients.

**Table 6:** Clinical evolution.

	Overall patients(n = 113)	Patients with allergic disorders (n = 24)	Other patients(n = 89)	Intergroup comparison values
Recovery until hospital discharge (days)	12.70 ± 9.91	12.95 ± 11.437	12.63 ± 9.51	P = 0.895^1^
Admission to the intensive care unit (yes/no)	11/102	1/23	10/79	P = 0.300^2^
Exitus (yes/no)	20/93	3/21	17/72	P = 0.452^2^

^1^T-test for independent samples.

^2^Pearson’s Chi-square test.

To analyze the days of recovery until hospital discharge we removed from the analyses patients who died, we stratified patients according to the median value (nine days) and we analyzed the putative impact of all covariates by using linear or logistic regression as appropriate. Results for univariate analyses are summarized in **Supplemental Table S1**. For each category of covariates, we performed a multiple regression analysis including any factor with a P-value equal to or lower than 0.300 in any of the groups studied and we carried out backward regression analyses. **Table 7** shows significant findings for multiple regression analyses and intergroup comparison values, and **Supplemental Table S2** shows the effect sizes for the covariates described in **Table 7**. Age was strongly correlated with days of recovery until hospital discharge. Patients with a long hospital stay had a mean age of about ten years more than that of patients with shorter hospital stays. This difference was observed in all subgroups of patients, although the statistical significance was weaker in patients with antecedents of allergy because of the smaller sample size. Antecedents of comorbidities other than allergy were also related to longer hospitalization times, although the findings were significant in overall patients only. Multiple regression analyses pointed to dyslipidemia, hypertension, and prothrombotic disorders as the parameters most associated with longer hospitalization, but T-test analyses do not show a major effect of any of these factors. Regression analyses also show statistically significant associations with the use of inhaled corticosteroids, and with the use of Angiotensin-II-receptor antagonists, although T-tests did not confirm any significant effect for these parameters (**Supplemental Table S2**). Regarding symptoms, multiple regression analyses suggest an association of shorter hospital stay in patients with musculoskeletal pain, although the differences in hospitalization days were small, and not significant according to T-test analyses. Regarding laboratory and clinical findings, oxygen saturation at hospital admission was a strong predictor of duration of the hospitalization time, with patients with lower saturation values tending to have longer hospitalization times (P = 0.001), although this difference was absent in patients with antecedents of allergy. The rest of the laboratory findings that were significant after multiple regression analyses (**Table 7**) were not significant in the T-test analyses. The absence of radiological findings was related to shorter (by about half) hospital stays (P = 0.023). Finally, treatment with corticosteroids after admission was consistently related to longer hospitalization times for all subgroups of patients.

**Table 7:** Significant findings after multiple regression analyses of covariates with recovery until hospital discharge, stratified by median value.

	Overall patients(n = 93). P value.	Asthma and allergy patients (n = 21). P value.	Other patients(n = 72). P value.
Age	0.002	n.s.	0.013
Comorbidity excluding asthma or allergy	0.015	n.s.	n.s.
Antecedents of dyslipidemia	n.s.	0.025	n.s.
Antecedents of hypertension	n.s.	n.s.	0.030
Antecedents of prothrombotic disorders	n.s.	0.010	0.050
Drugs used in allergic diseases			
Inhaled corticosteroids	0.051	0.033	0.005
Drugs used in cardiovascular diseases			
Angiotensin-II-receptor antagonists	n.s.	0.043	n.s.
Symptoms			
Musculoskeletal pain	0.012	n.s.	0.021
Laboratory and clinical findings			
Oxygen saturation	0.007	n.s.	0.006
Leukocytes per microliter	n.s	n.s	0.034
Eosinophils per microliter	0.006	n.s.	n.s.
D-dimer, ng/mL	0.006	n.s.	n.s
Ferritin, ng/mL	n.s	n.s	0.002
Radiological findings			
No radiological findings	0.005	n.s.	0.002
Drug therapy after admission			
Lopinavir plus ritonavir	0.032	n.s.	n.s.
Corticosteroids	0.001	0.033	0.001

To analyze admission to the ICU, we assessed the putative impact of all covariates by linear or logistic regression as appropriate. Results for univariate analyses are summarized in **Supplemental Table S3** and significant findings in multivariate analyses are shown in **Table 8**. The effect sizes for covariates with significant associations are summarized in **Supplemental Table S4**. It should be noted that only one patient with antecedents of allergic diseases was admitted to the ICU and therefore covariate analyses in this subgroup of patients are not informative. Age, gender, and antecedents of comorbidities were not related to admission to the ICU. Regarding previous drug therapy, patients receiving calcium-channel blockers before hospital admission had a statistically significant increased risk of admission to ICU (P = 0.005). The previous use of proton pump inhibitors was also related to increased risk of admission to ICU in overall patients (P = 0.017). Since the consumption of these drugs might be related to age, we performed multiple regression analyses including age and consumption of calcium channel blockers, and the results indicated that consumption of calcium channel blockers remained significant (P = 0.013). Multiple regression including age and proton pump inhibitors indicated that the use of proton pump inhibitors was the most significant factor (P = 0.014). Regarding symptoms at hospital admission, overall patients presenting with headaches had a significantly increased risk (RR = 4.80, P = 0.020). The laboratory findings at hospital admission most associated with admission to ICU were low oxygen saturation (P = 0.002), high leukocyte count (P = 0.011), and high D-dimer concentration (P = 0.005). Bilateral radiological findings at hospital admission were also strongly associated to ICU admission (RR = 6.87, P = 0.025), and tocilizumab use after admission was also related to admission to ICU (RR = 12.34, P = 0.001).

**Table 8:** Significant findings after multiple regression analyses of covariates with admission to the intensive care unit.

	Overall patients(n = 113)	Patients with allergic disorders (n = 24)	Other patients(n = 89)
Drugs used in cardiovascular diseases			
Calcium channel blockers	0.010	n.s.	0.013
Other drugs			
Proton pump inhibitors	0.031	n.s.	0.086
Symptoms			
Headache	0.041	n.s.	0.573
Laboratory and clinical findings			
Oxygen saturation	0.001	–	0.001
Leukocytes per microliter	0.002	–	n.s.
D-dimer, ng/mL	0.001	–	0.001
Radiological findings			
Bilateral radiological findings	0.006	n.s.	0.001
Drug therapy after admission			
Tocilizumab	0.001	n.s.	0.001

Results of univariate analyses for mortality risk are summarized in **Supplemental Table S5**, significant findings after multivariate analyses are shown in **Table 9** and the effect sizes for covariates with significant associations are summarized in **Supplemental Table S6**. Age was strongly related to mortality risk (P = 0.001 for overall patients). For patients with antecedents of allergy the mean age of patients who died was 67 years whereas the mean age of patients who survived was 58 years. For patients without antecedents of allergy diseases, the mean ages were 82 and 67 years for patients who died and survived, respectively. Antecedents of cerebrovascular diseases were related to an increased mortality risk ratio equal to 10.5 (P = 0.034). This effect was observed in all subgroups of patients. Increased mortality rates were observed in patients with previous use of oral anticoagulants (P = 0.009), non-steroidal anti-inflammatory drugs (P = 0.043), selective serotonin reuptake inhibitors (P = 0.003), antiepileptic drugs (P = 0.012), and proton pump inhibitors (P = 0.008). Since all these drug therapies could be related to age, we performed multiple regression analyses including age plus each of the previously mentioned drug therapies. The results indicate that the statistical significance was lost for non-steroidal anti-inflammatory drugs (P = 0.143), antiepileptic drugs (P = 0.171), and proton pump inhibitors (P = 0.072). However, the significance remained for oral anticoagulants (P = 0.048), and selective serotonin reuptake inhibitors (P = 0.038). Patients with high Interleukin-6 concentrations have increased mortality risk (P = 0.001) as shown in **Supplemental Table S6**.

**Table 9:** Significant findings after multiple regression analyses of covariates with mortality.

	Overall patients(n = 93). P value.	Asthma and allergy patients (n = 21). P value.	Other patients(n = 72). P value.
Age	0.001	n.s.	0.013
Comorbidity excluding asthma or allergy			
Antecedents of cerebrovascular disorders	0.008	–	0.026
Drugs used in cardiovascular diseases			
Oral anticoagulants	0.007	n.s.	n.s.
Platelet Antiaggregants	0.034	n.s.	n.s.
Pain relief medications			
Non-steroidal anti-inflammatory drugs	0.047	n.s.	0.052
Psychiatric medications			
Selective serotonin reuptake inhibitors	0.004	n.s.	0.022
Drug used in neurological diseases			
Antiepileptic drugs	0.028	n.s.	0.022
Other drugs			
Proton pump inhibitors	0.011	n.s.	n.s.
Laboratory and clinical findings			
Oxygen saturation	0.007	n.s.	0.017
Lymphocytes	0.034	n.s.	n.s.
Eosinophils	0.010	n.s.	0.021
Interleukin-6	0.001	n.s.	0.001
Drug therapy after admission			
Lopinavir plus ritonavir	0.011	n.s.	0.005

## Discussion

Although previous studies have suggested that patients with asthma or allergy are under-represented among patients with Covid-19 (Li et al., 2020; Zhang et al., 2020), these findings were obtained in Oriental individuals, which have a low frequency for these disorders, and so need to be replicated in different human populations. Of the 113 patients with Covid-19 analyzed in our study, 24 had antecedents of allergic disorders, seven patients (6.2%) suffered from asthma and eight patients suffered from rhinitis (7.1%). This asthma percentage, similar to that observed in the Spanish general population (Quirce et al., 2011), is much higher than the percentages in two series of Chinese patients, which reported 0 and 0.9% of asthmatic patients, respectively (Li et al., 2020; Zhang et al., 2020). The percentage of asthmatic patients in this study is similar to previously published Western studies (Lechien et al., 2020; Richardson et al., 2020), although smaller than a report from the USA Center for Disease Control and Prevention (Garg et al., 2020).

Most Covid-19 patients (82.30%) have different comorbidities, apart from allergy or asthma, as described in previously published series (Chen et al., 2020; Goyal et al., 2020; Guan et al., 2020b; Huang et al., 2020; Lechien et al., 2020; Richardson et al., 2020; Wang et al., 2020a; Wang et al., 2020b; Zhao et al., 2020). The most frequent comorbidities in our study group of overall patients were hypertension, heart diseases, dyslipidemia, type II diabetes, and renal diseases. Hypertension is one of the main comorbidities described in hospitalized Covid-19 patients in different countries (Huang et al., 2020; Goyal et al., 2020; Guan et al., 2020b; Guzik et al., 2020; Richardson et al., 2020; Wang et al., 2020a; Wang et al., 2020b). We found a similar percentage of patients with hypertension compared to Covid-19 Western patients series (Goyal et al., 2020; Richardson et al., 2020), this frequency being superior to that reported in Chinese patient series (Guan et al., 2020b; Wang et al., 2020a; Wang et al., 2020b). No significant differences in comorbidities were found between the overall patient group, patients without allergic disorders, and patients with allergic disorders.

Collected data regarding drug treatment before hospital admission for all of the patients included 33 drug classes. As was to be expected, statistically significant differences were observed between patients with allergic disorders and the other subgroup of patients for ICS consumption (**Table 2**). A study has been published concluding that the use of ICS brings about a lower expression of ACE2 and transmembrane protease serine 2 (TMPRSS2), and it has been speculated that this might decrease susceptibility to SARS-CoV-2 infection, hence decreasing morbidity produced by Covid-19 (Peters et al., 2020). These findings support the recommendation of continuing ICS therapy in asthma patients (Maes et al., 2020). Nevertheless, a systematic review has been published about ICS and Covid-19 concluding that there is no evidence for a beneficial or a detrimental effect of their use for SARS-CoV-2 infection (Halpin et al., 2020b). In our study group, we did not observe any impact of the use of ICS in the clinical outcome. With regard to ACE2 and allergy patients, studies with controversial results have been published, some showing that allergy respiratory patients have reduced ACE2 expression in airway cells (Jackson et al., 2020) or indicating that IL-13 in allergic rhinitis and asthma patients significantly reduced ACE2 (Bradding et al., 2020). Nevertheless, other authors have observed the opposite results in an experimental study (Sajuthi et al., 2020). Our findings do not support any major effect of the use of ACE inhibitors or angiotensin II antagonists in any of the clinical outcomes determined in this study.

The most frequent recorded symptoms in the overall patients’ group were fever (71.7%), dry cough, and dyspnea (**Table 3**). Fever is the most frequently reported symptom in most of the series previously published, with frequencies ranging from 43.8% (Guan et al., 2020a) to 98.6% (Wang et al., 2020b). Zhao et al. (2020) and Goyal et al. (2020) have described percentages of patients presenting with fever similar to those reported here (64.8 and 77.1% in Hubei province and New York, respectively). No significant differences in symptoms were found between overall patients, patients with allergic disorders, and patients without allergic disorders.

Laboratory findings are summarized in **Table 4**. We observed elevated medium values for those parameters that indicate an inflammatory systemic response, such as C-reactive protein, D-dimer, ferritin, and Il-6. Lymphopenia has been described in several studies (Huang et al., 2020; Li et al., 2020; Zhang et al., 2020; Zhao et al., 2020). The mean number of lymphocytes in our patients was 1,891.07 per microliter. This number is highly influenced by the fact that 2 patients in the overall group suffered from chronic lymphatic leukemia, with a number of lymphocytes equal to 4,560 and 86,630 per microliter, respectively. To overcome this distortion we calculated the median of the number of lymphocytes in our series, which resulted in 1,040 lymphocytes per microliter, matching the previously published values.

The mean number of blood eosinophils in the overall group was 44.28 per microliter (0–500 per microliter). Some series of hospitalized Covid-19 patients have described low eosinophils blood count (Liu F. et al., 2020; Du Y. et al., 2020; Zhang et al., 2020). A correlation has been shown between blood eosinophils and lymphocyte counts (Niedzwiedz et al., 2020) and eosinopenia has been proposed as a prognostic indicator of a more severe Covid-19 outcome (Lindsley et al., 2020). Normal eosinophil values had been observed after lopinavir treatment (Liu F. et al., 2020). Eosinophil count was not a predicting factor in our study, but high leukocyte count was a predicting factor for ICU admission (P = 0.011, **Supplemental Table S4**).

Most patients presented radiological findings of pneumonia in the chest X-Ray and/or CT scan, habitually interstitial or alveolar-interstitial infiltrates, the bilateral radiological findings being more frequent (40.70%) than unilateral findings (23.89%). No significant differences in the laboratory or radiological findings were found between the overall patient group, patients without allergic disorders and patients with allergic disorders. However, according to our results, radiological findings at hospital admission were good predictors of recovery time and ICU admission.

In addition to the support treatment used in Covid-19 hospitalized patients, the most frequent “specific” treatments used in the overall patients were hydroxychloroquine, azithromycin, and lopinavir plus ritonavir. Some patients also received systemic corticosteroids, interferon beta, and tocilizumab. All patients were also treated with subcutaneous low molecular weight heparin, usually enoxaparin, as recommended by the hospital treatment protocol. Hydroxychloroquine has been widely used in Covid-19 patients for weeks, based on a low-evidence open-label non-randomized study in which efficacy, when used alone or combined with azithromycin, was observed (Gautret et al., 2020). Very recently an international multicentre observational study has been published including 96,032 patients. The authors conclude that they were not able to confirm the benefit of hydroxychloroquine treatment used alone or associated with a macrolide in Covid-19 hospitalized patient outcomes. Moreover, treatment with hydroxychloroquine was associated with increased mortality and increased frequency of arrhythmias (Mehra et al., 2020a). Due to these results, on May 27^th^, 2020 the WHO recommended not to use hydroxychloroquine in Covid-19 patients and interrupted the global clinical trial with hydroxychloroquine (^4^). Nevertheless, three of the four authors of the study published by Mehra et al. (2020a) have requested this paper to be retracted due to doubts about the veracity of the primary data sources (Mehra et al., 2020b). Moreover, on June 3^rd^, WHO has recommended to include again hydroxichloroquine in the global clinical trial (^4^).

In our study, no significant differences in drug therapy received after hospital admission were found between overall patients, patients with allergic disorders, and patients without allergic disorders.

Concerning clinical evolution, we analyzed time to recover until hospital discharge, admission to the ICU, and mortality rate. There is a previous study which concludes that among Covid-19 hospitalized patients who developed severe respiratory symptoms requiring mechanical ventilation, asthma was associated with a significantly longer intubation time (Mahdavinia et al., 2020). In the present study, no significant differences in any of those variables were found between overall patients, patients with allergic disorders, and patients without allergic disorders.

Patient age was strongly correlated with days of recovery until hospital discharge and those patients with shorter hospital stay were ten years younger than patients with a longer hospital stay. This difference was observed in all subgroups of patients, although the statistical significance was weaker in patients with allergic disorders because of the smaller sample size. Age was also a mortality predictor, as shown in **Supplemental Table S6**.

Antecedents of comorbidities other than allergy were also related to longer hospitalization times, although the findings were significant in overall patients only. These results are in concordance with a previously published study in which authors observed that patients with comorbidity had worse clinical outcomes compared with patients without comorbidities (Guan et al., 2020a). Longer hospitalization times were also significantly related to low oxygen saturation at hospital admission and with treatment with corticosteroids. Also, the absence of radiological findings was related to shorter (by about half) hospital stays.

In our study, admission to the ICU was not related to age, gender, nor antecedents of comorbidities. In contrast, ICU admission was related to the previous use of calcium-channel blockers and proton pump inhibitors, low oxygen saturation, high leukocyte count, high D-dimer concentration, bilateral radiological findings, and headaches. Wang et al. (2020a) have also reported high leukocyte count and high D-dimer in patients admitted to the ICU, and Phua et al. have described low oxygen saturation and the presence of bilateral radiological findings in these patients (Phua et al., 2020). Tocilizumab has been proposed to be used in severe and critical patients in the context of a cytokine release syndrome (Liu B. et al., 2020). In our study tocilizumab use was high in patients admitted to the ICU.

Regarding calcium-channel blockers, a study has been published showing that nifedipine and amlodipine were found to be associated with decreased risk of mortality and mechanical ventilation in elderly Covid-19 hospitalized patients (Solaimanzadeh, 2020). The putative association between calcium-channel blockers and ICU admission observed in the present study deserves further investigation, as does the association of the previous use of proton pump inhibitors with a higher frequency of ICU admission observed in this study. We initially hypothesized that the latter association could be related to age, but multivariate analyses indicated that the association remained statistically significant regardless of age. As far as we are aware, there are no specific published studies investigating proton pump inhibitors and Covid-19.

In our cohort, age was strongly related to mortality. Increased mortality was also related to antecedents of cerebrovascular diseases and with the previous use of oral anticoagulant agents and selective serotonin reuptake inhibitors. Old age as a risk factor for mortality has been described elsewhere (Du R. H. et al., 2020; Li et al., 2020; Ruan et al., 2020; Zhou et al., 2020) and Du et al. have also described cerebrovascular diseases as a risk factor for mortality (Du R. H. et al., 2020).

Our study has some limitations. Data were obtained retrospectively from electronic clinical histories. Some patients with antecedents of allergic disorders were studied in Allergy or Pneumology departments, but in many cases, allergy antecedents were self-reported by patients, although all these patients had a previous clinical diagnosis of allergic diseases. The sample size of patients with antecedents of allergic disorders prevented us from making specific statistical analyses with subcategories of allergic diseases. We acknowledge that the population studied here might not reflect the whole population in Spain and that the sample size is too low to assess the effect of antecedents of allergic diseases in mortality. Finally, the atopic status was determined by clinical examination and antecedents, but type 2 cytokines were not tested for during hospitalization.

In summary, the frequency of patients with antecedents of allergic diseases is similar in our study group to the general population, but these patients are younger and have fewer comorbidities than the rest of hospitalized Covid-19 patients. Clinical outcomes are similar between patients with antecedents of allergic diseases and the rest of patients, and previous anti-allergic drug therapy is not a major factor determining clinical evolution.

## Data Availability Statement

All datasets presented in this study are included in the article/**Supplementary Material**.

## Ethics Statement

The studies involving human participants were reviewed and approved by The Research Ethics Committee of the Badajoz Health Area. Written informed consent for participation was not required for this study in accordance with the national legislation and the institutional requirements because the data was collected as part of routine clinical care.

## Author Contributions

All authors contributed to the article and approved the submitted version. JG-M and CC-D wrote the first draft of the manuscript. EG-M and JA wrote sections of the manuscript and performed statistical analysis. JG-M acquired data.

## Funding

This work was supported in part by Grants RETICS RD16/0006/0004 (ARADyAL), PI15/00303, and PI18/00540 from Fondo de Investigación Sanitaria, Instituto de Salud Carlos III, Madrid, Spain and GR18145 and IB16170 from Junta de Extremadura, Mérida, Spain. Partially funded with FEDER funds.

## Conflict of Interest

The authors declare that the research was conducted in the absence of any commercial or financial relationships that could be construed as a potential conflict of interest.

## Footnotes

1 World Health Organization (WHO). Naming the coronavirus disease (COVID-19) and the virus that causes it. Available from: https://www.who.int/emergencies/diseases/novel-coronavirus-2019/technical-guidance/naming-the-coronavirus-disease-(covid-2019)-and-the-virus-that-causes-it [Accessed May 22, 2020]

2 Spanish Ministry of Health. Available from: https://www.mscbs.gob.es/en/profesionales/saludPublica/ccayes/alertasActual/nCov-China/situacionActual.htm. [Accessed June 7, 2020]

3 CDC COVID-19 Response Team. Geographic Differences in COVID-19 Cases, Deaths, and Incidence - United States, February 12-April 7, 2020. MMWR Morb Mortal Wkly Rep (2020) 69:465–471. doi:10.15585/mmwr.mm6915e4

4 “Solidarity” clinical trials for COVID-19 treatments. Available from: https://www.who.int/emergencies/diseases/novel-coronavirus-2019/global-research-on-novel-coronavirus-2019-ncov/solidarity-clinical-trial-for-covid-19-treatments [Accessed June 7, 2020]
