# Supplementary material for: Outcomes and Laboratory and Clinical Findings of Asthma and Allergic Patients Admitted With Covid-19 in a Spanish University Hospital
Source: Front Pharmacol. 2020 Sep 16;11:570721. doi: 10.3389/fphar.2020.570721 (PMC7525217; doi:10.3389/fphar.2020.570721)
Supplement: Supplementary file 1 [file DataSheet_1.docx]

Supplemental Table S1. Univariate associations with recovery time until hospital discharge.

|  | Overall patients  (n=93) | Asthma and allergy patients (n=21) | Other patients  (n=72) |
| --- | --- | --- | --- |
| Age & gender |  |  |  |
| Age | 0.002 | 0.066 | 0.015 |
| Gender | 1.000 | 0.530 | 0.702 |
| Antecedents of allergic diseases | 0.635 |  |  |
| Drug allergy | 0.419 |  |  |
| Asthma and rhinitis | 0.543 |  |  |
| Seasonal allergy | 0.950 |  |  |
| Urticaria | 0.950 |  |  |
| Comorbidity excluding asthma or allergy | 0.033 | 0.115 | 0.146 |
| Antecedents of hypertension | 0.038 | 0.890 | 0.030 |
| Antecedents of heart diseases | 0.469 | 0.529 | 0.242 |
| Antecedents of dyslipidemia | 0.135 | 1.000 | 0.086 |
| Antecedents of type II diabetes | 0.175 | 1.000 | 0.112 |
| Antecedents of renal diseases | 0.306 | -- | 0.242 |
| Antecedents of thrombotic disorders | 0.438 | 0.123 | 0.058 |
| Antecedents of respiratory diseases^3^ | 0.288 | 0.999 | 0.658 |
| Antecedents of cerebrovascular diseases | 0.469 | 1.000 | 0.764 |
| Antecedents of cognitive impairment | 0.366 | 0.999 | 0.840 |
| Antecedents of psychiatric diseases | 0.366 | 1.002 | 0.591 |
| Antecedents of hormonal disorders | 0.610 | 1.000 | 0.857 |
| Antecedents of neurological diseases | 0.579 | 1.000 | 0.885 |
| Antecedents of digestive diseases | 0.391 | 1.000 | 0.181 |
| Antecedents of neoplastic diseases | 0.469 | -- | 0.505 |
| Antecedents of morbid obesity | 0.543 | 0.999 | 0.857 |
| Antecedents of anaemia | 0.174 | 1.000 | 0.268 |
| Drugs used in allergic diseases | 0.151 | 0.135 | 0.403 |
| Inhaled corticosteroids | 0.193 | 0.060 | 0.840 |
| Inhaled Beta-2 agonist | 0.288 | 0.135 | 0.764 |
| Inhaled Anticholinergics | 0.099 | 0.485 | 0.999 |
| Antihistamine drugs | 0.370 | 0.944 | 0.999 |
| Anti-leukotrienes agents | 1.000 | 1.000 | -- |
| Omalizumab | 1.000 | 1.000 | -- |
| Drugs used in cardiovascular diseases |  |  |  |
| Statins | 0.200 | 0.529 | 0.280 |
| Angiotensin-II-receptor antagonists | 0.200 | 0.332 | 0.075 |
| Diuretics | 1.000 | 0.999 | 0.692 |
| Platelet Antiaggregants | 0.638 | 0.485 | 0.367 |
| Beta-blockers | 0.837 | 0.529 | 0.557 |
| Calcium channel blockers | 0.468 | 0.944 | 0.467 |
| Oral anticoagulants | 0.194 | 0.485 | 0.247 |
| Angiotensin-converting enzyme inhibitors | 0.601 | 1.000 | 0.896 |
| Other vasodilators | 0.194 | -- | 0.212 |
| Pain relief medications |  |  |  |
| Non-steroidal anti-inflammatory drugs | 0.804 | 0.182 | 0.655 |
| Opiates | 0.601 | 0.597 | 0.328 |
| Psychiatric medications |  |  |  |
| Benzodiazepines | 0.968 | 0.597 | 0.938 |
| Selective serotonin reuptake inhibitor | 0.127 | 0.597 | 0.056 |
| Antipsychotic agents | 0.785 | 1.000 | 0.857 |
| Other antidepressants | 0.999 | 0.999 | 0.999 |
| Drug used in neurological diseases |  |  |  |
| Anti-Parkinson Drugs | 0.615 | -- | 0.645 |
| Antiepileptic drugs | 0.950 | 1.000 | 1.000 |
| Other drugs |  |  |  |
| Proton pump inhibitors | 0.619 | 0.916 | 0.702 |
| Oral antihyperglycemic drugs | 0.576 | 0.999 | 0.247 |
| Antibiotics | 0.898 | 1.000 | 0.554 |
| Levothyroxine | 0.720 | -- | 0.764 |
| Antiandrogens | 0.099 | -- | 0.106 |
| Oral Anticholinergics | 0.999 | -- | 0.999 |
| Adrenergic alpha 1-antagonists | 0.929 | -- | 0.885 |
| Immunosuppressants | 0.517 | 1.000 | 0.919 |
| Oral corticosteroids | 1.000 | -- | 1.000 |
| Symptoms |  |  |  |
| Fever | 0.507 | 0.890 | 0.494 |
| Dry cough | 0.382 | 0.890 | 0.361 |
| Dyspnea | 0.439 | 0.758 | 0.446 |
| Overall discomfort | 0.840 | 0.182 | 0.361 |
| Asthenia | 0.638 | 1.000 | 0.729 |
| Diarrhoea | 0.400 | 0.279 | 0.714 |
| Musculoskeletal pain | 0.012 | 0.332 | 0.021 |
| Anosmia | 0.999 | 1.000 | 0.999 |
| Ageusia | 0.999 | 1.000 | 0.999 |
| Headache | 0.293 | -- | 0.268 |
| Nauseas and/or vomits | 0.517 | 0.944 | 1.000 |
| Odynophagia | 0.293 | 0.944 | 0.999 |
| Exanthema | 1.000 | -- | 1.000 |
| Laboratory and clinical findings |  |  |  |
| Temperature | 0.323 | 0.032 | 0.995 |
| Oxygen saturation | 0.001 | 0.774 | 0.001 |
| Leukocytes per microliter | 0.089 | 0.355 | 0.154 |
| Lymphocytes per microliter | 0.091 | 0.458 | 0.140 |
| Eosinophils per microliter | 0.240 | 0.735 | 0.157 |
| Platelet count per microliter | 0.070 | 0.606 | 0.084 |
| Aspartate Aminotransferase, IU/L | 0.820 | 0.184 | 0.463 |
| Alanine Aminotransferase, IU/L | 0.517 | 0.060 | 0.918 |
| Lactate dehydrogenase, IU/L | 0.001 | 0.682 | 0.001 |
| Creatine Phosphokinase, IU/L | 0.125 | 0.274 | 0.261 |
| C-reactive protein, mg/L | 0.005 | 0.558 | 0.005 |
| Creatinine, mg/dL | 0.267 | 0.765 | 0.216 |
| D-dimer, ng/mL | 0.233 | 0.407 | 0.267 |
| Ferritin, ng/mL | 0.268 | 0.423 | 0.091 |
| Procalcitonin, ng/mL | 0.678 | 0.471 | 0.398 |
| Interleukin-6, pg/mL | 0.421 | 0.300 | 0.666 |
| Radiological findings |  |  |  |
| No radiological findings | 0.012 | 0.332 | 0.021 |
| Unilateral radiological findings | 0.337 | 0.485 | 0.156 |
| Bilateral radiological findings | 0.004 | 0.758 | 0.002 |
| Drug therapy after admission |  |  |  |
| Hydroxychloroquine | 0.391 | 0.944 | 0.313 |
| Azithromycin | 0.860 | 0.999 | 0.370 |
| Lopinavir plus ritonavir | 0.043 | 0.801 | 0.032 |
| Corticosteroids | 0.001 | 0.060 | 0.001 |
| Interferon beta | 0.999 | 1.000 | 0.999 |
| Tocilizumab | 0.999 | -- | 0.999 |

Supplemental Table S2. Effect sizes for covariates related with recovery until hospital discharge.

|  | Overall patients  (n=93). P value. | Asthma and allergy patients (n=21). P value. | Other patients  (n=72). P value. |
| --- | --- | --- | --- |
| Age (years). Patients with long hospital stay. | 69.46 ± 12.09 | 65.00 ± 12.54 | 70.63 ± 11.86 |
| Age (years). Patients with short hospital stay. | 59.48 ± 17.55 | 51.82 ± 17.67 | 62.03 ± 17.01 |
| T-test (P) | 0.002 | 0.066 | 0.015 |
| Antecedents of any comorbidity excluding asthma or allergy. Days ± SD. | 13.40 ± 9.51 | 16.69 ± 13.05 | 12.68 ± 8.51 |
| No antecedents of any comorbidity excluding asthma or allergy. Days ± SD. | 8.30 ± 6.53 | 6.88 ± 3.64 | 9.25 ± 7.92 |
| T-test (P) | 0.027 | 0.053 | 0.203 |
| Antecedents of dyslipidemia. Days ± SD. | 15.71 ± 10.59 | 7.00 | 16.15 ± 10.67 |
| No antecedents of dyslipidemia. Days ± SD. | 11.82 ± 9.61 | 13.25 | 11.27 ± 8.77 |
| T-test (P) | 0.114 | 0.606 | 0.050 |
| Antecedents of hypertension. Days ± SD. | 14.57 ± 10.95 | 18.67 ± 17.28 | 13.95 ± 9.85 |
| No antecedents of hypertension. Days ± SD. | 10.87 ± 8.51 | 10.67 ± 7.77 | 10.97 ± 8.95 |
| T-test (P) | 0.072 | 0.152 | 0.188 |
| Antecedents of thrombotic disorders. Days ± SD. | 13.17 ± 13.08 | 21.00 ± 17.76 | 7.57 ± 3.95 |
| No antecedents of thrombotic disorders. Days ± SD. | 12.63 ± 9.46 | 10.44 ± 7.86 | 13.17 ± 9.79 |
| T-test (P) | 0.862 | 0.070 | 0.140 |
| Use of inhaled corticosteroids. Days ± SD. | 13.23 ± 9.63 | 13.00 ± 7.29 | 13.43 ± 11.87 |
| No use of inhaled corticosteroids. Days ± SD. | 12.61 ± 10.02 | 12.93 ± 12.95 | 12.54 ± 9.33 |
| T-test (P) | 0.836 | 0.991 | 0.816 |
| Use of angiotensin-II-receptor antagonists. Days ± SD. | 15.66 ± 12.63 | 18.25 ± 21.19 | 15.24 ± 11.35 |
| No use of angiotensin-II-receptor antagonists. Days ± SD. | 11.36 ± 8.17 | 11.71 ± 8.40 | 11.23 ± 8.17 |
| T-test (P) | 0.052 | 0.315 | 0.089 |
| Symptoms |  |  |  |
| Musculoskeletal pain. Days ± SD. | 8.80 ± 6.55 | 10.50 ± 11.79 | 8.18 ± 4.09 |
| No Musculoskeletal pain. Days ± SD. | 13.45 ± 10.30 | 13.53 ± 11.64 | 13.43 ± 10.01 |
| T-test (P) | 0.096 | 0.645 | 0.093 |
| Laboratory and clinical findings |  |  |  |
| Oxygen saturation (%). Patients with long hospital stay | 92.3 ± 4.32 | 94.00 ± 2.98 | 91.72 ± 4.56 |
| Oxygen saturation (%). Patients with short hospital stay | 95.2 ± 3.42 | 94.45 ± 4.03 | 95.48 ± 3.18 |
| T-test (P) | 0.001 | 0.774 | 0.001 |
| Leukocytes per microliter. Patients with long hospital stay | 8063.13 ± 3504.56 | 8720.00 ± 4767.85 | 7890.23 ± 3150.33 |
| Leukocytes per microliter. Patients with short hospital stay | 6800.00 ± 3533.20 | 6956 ± 3734.85 | 6747.88 ± 3521.98 |
| T-test (P) | 0.089 | 0.355 | 0.154 |
| Eosinophils per microliter. Patients with long hospital stay | 38.96 ± 60.54 | 37.00 ± 64.99 | 39.47 ± 60.22 |
| Eosinophils per microliter. Patients with short hospital stay | 58.95 ± 98.01 | 29.09 ± 35.59 | 69.22 ± 110.45 |
| T-test (P) | 0.240 | 0.735 | 0.157 |
| D-dimer, ng/mL. Patients with long hospital stay | 6279.43 ± 26325.16 | 1340.67 ± 1580.04 | 7480.76 ± 29293.88 |
| D-dimer, ng/mL. Patients with short hospital stay | 1322.00 ± 1999.54 | 917.27 ± 464.99 | 1470.40 ± 2314.16 |
| T-test (P) | 0.233 | 0.407 | 0.276 |
| Ferritin, ng/mL. Patients with long hospital stay | 1041.97 ± 1186.61 | 770.88 ± 657.16 | 1114.27 ± 1290.95 |
| Ferritin, ng/mL. Patients with short hospital stay | 784.45 ± 782.94 | 1145.30 ± 1141.00 | 655.57 ± 583.62 |
| T-test (P) | 0.268 | 0.423 | 0.091 |
| Radiological findings |  |  |  |
| No radiological findings. Days ± SD. | 7.40 ± 3.38 | 7.50 ± 2.89 | 7.36 ± 3.67 |
| Radiological findings. Days ± SD. | 13.72 ± 10.43 | 14.24 ± 12.35 | 13.57 ± 9.94 |
| T-test (P) | 0.023 | 0.301 | 0.045 |
| Drug therapy after admission |  |  |  |
| Lopinavir plus ritonavir. Days ± SD. | 13.47 ± 9.78 | 11.83 ± 7.90 | 13.89 ± 10.25 |
| No use of lopinavir plus ritonavir. Days ± SD. | 11.43 ± 10.14 | 14.44 ± 15.38 | 10.38 ± 7.72 |
| T-test (P) | 0.340 | 0.617 | 0.134 |
| Corticosteroids. Days ± SD. | 17.11 ± 7.95 | 18.67 ± 10.01 | 16.67 ± 7.49 |
| No use of corticosteroids. Days ± SD. | 10.89 ± 10.12 | 10.67 ± 11.46 | 10.96 ± 9.82 |
| T-test (P) | 0.005 | 0.152 | 0.020 |

Supplemental Table S3. Univariate associations with the admission to the intensive care unit.

|  | Overall patients  (n=113) | Patients with allergic disorders (n=24) | Other patients  (n=89) |
| --- | --- | --- | --- |
| Age & gender |  |  |  |
| Age | 0.832 | 0.616 | 0.714 |
| Gender | 0.133 | 0.999 | 0.275 |
| Antecedents of allergic diseases | 0.320 |  |  |
| Drug allergy | 0.999 |  |  |
| Asthma and rhinitis | 0.999 |  |  |
| Seasonal allergy | 0.976 |  |  |
| Urticaria | 0.320 |  |  |
| Comorbidity excluding asthma or allergy | 0.853 | 0.999 | 0.435 |
| Antecedents of hypertension | 0.069 | 0.998 | 0.179 |
| Antecedents of heart diseases | 0.355 | 0.998 | 0.614 |
| Antecedents of dyslipidemia | 0.314 | 0.998 | 0.887 |
| Antecedents of type II diabetes | 0.666 | 0.999 | 0.750 |
| Antecedents of renal diseases | 0.387 | -- | 0.547 |
| Antecedents of thrombotic disorders | 0.522 | 0.998 | 0.999 |
| Antecedents of respiratory diseases | 0.616 | 0.998 | 0.733 |
| Antecedents of cerebrovascular diseases | 0.792 | 0.999 | 0.810 |
| Antecedents of cognitive impairment | 0.999 | 0.999 | 0.999 |
| Antecedents of psychiatric diseases | 0.999 | 0.999 | 0.999 |
| Antecedents of hormonal disorders | 0.940 | 1.000 | 0.896 |
| Antecedents of neurological diseases | 0.976 | 0.999 | 0.906 |
| Antecedents of digestive diseases | 0.976 | 1.000 | 0.990 |
| Antecedents of neoplastic diseases | 0.976 | -- | 0.896 |
| Antecedents of morbid obesity | 0.999 | 0.999 | 0.999 |
| Antecedents of anaemia | 0.999 | 1.000 | 0.999 |
| Drugs used in allergic diseases | 0.898 | 0.999 | 0.896 |
| Inhaled corticosteroids | 0.688 | 0.998 | 0.906 |
| Inhaled Beta-2 agonist | 0.616 | 0.999 | 0.666 |
| Inhaled Anticholinergics | 0.207 | 0.998 | 0.531 |
| Antihistamine drugs | 0.999 | 0.999 | 0.999 |
| Anti-leukotrienes agents | 1.000 | 1.000 | -- |
| Omalizumab | 1.000 | 1.000 | -- |
| Drugs used in cardiovascular diseases |  |  |  |
| Statins | 0.425 | 0.998 | 0.716 |
| Angiotensin-II-receptor antagonists | 0.116 | 0.999 | 0.105 |
| Diuretics | 0.214 | 0.999 | 0.169 |
| Platelet Antiaggregants | 0.314 | 0.999 | 0.682 |
| Beta-blockers | 0.794 | 0.999 | 0.985 |
| Calcium channel blockers | 0.010 | 0.999 | 0.010 |
| Oral anticoagulants | 0.314 | 0.999 | 0.682 |
| Angiotensin-converting enzyme inhibitors | 0.863 | 0.998 | 0.999 |
| Other vasodilators | 0.976 | 1.000 | 0.990 |
| Pain relief medications |  |  |  |
| Non-steroidal anti-inflammatory drugs | 0.281 | 0.998 | 0.597 |
| Opiates | 0.202 | 0.998 | 0.443 |
| Psychiatric medications |  |  |  |
| Benzodiazepines | 0.387 | 0.998 | 0.777 |
| Selective serotonin reuptake inhibitor | 0.176 | 0.998 | 0.419 |
| Antipsychotic agents | 0.999 | 0.999 | 0.999 |
| Other antidepressants | 0.677 | 0.999 | 0.531 |
| Drug used in neurological diseases |  |  |  |
| Anti-Parkinson Drugs | 0.563 | -- | 0.666 |
| Antiepileptic drugs | 0.999 | 1.000 | 0.999 |
| Other drugs |  |  |  |
| Proton pump inhibitors | 0.031 | 0.998 | 0.086 |
| Oral antihyperglycemic drugs | 0.666 | 0.999 | 0.682 |
| Antibiotics | 0.332 | 1.000 | 0.362 |
| Levothyroxine | 0.885 | -- | 0.986 |
| Antiandrogens | 0.207 | -- | 0.285 |
| Oral Anticholinergics | 0.999 | -- | 0.999 |
| Adrenergic alpha 1-antagonists | 0.041 | -- | 0.061 |
| Immunosuppressants | 0.442 | 1.000 | 0.391 |
| Oral corticosteroids | 0.205 | -- | 0.254 |
| Symptoms |  |  |  |
| Fever | 0.438 | 0.999 | 0.501 |
| Dry cough | 0.316 | 0.999 | 0.376 |
| Dyspnea | 0.805 | 0.999 | 0.478 |
| Overall discomfort | 0.281 | 0.998 | 0.597 |
| Asthenia | 0.965 | 1.000 | 0.912 |
| Diarrhoea | 0.387 | 0.999 | 0.201 |
| Musculoskeletal pain | 0.289 | 0.998 | 0.611 |
| Anosmia | 0.999 | 1.000 | 0.999 |
| Ageusia | 0.976 | 1.000 | 0.990 |
| Headache | 0.041 | -- | 0.391 |
| Nauseas and/or vomits | 0.999 | 0.999 | 0.999 |
| Odynophagia | 0.999 | 0.999 | 0.999 |
| Exanthema | 1.000 | -- | 1.000 |
| Laboratory and clinical findings |  |  |  |
| Temperature | 0.515 | -- | 0.399 |
| Oxygen saturation | 0.002 | -- | 0.010 |
| Leukocytes per microliter | 0.011 | 0.644 | 0.013 |
| Lymphocytes per microliter | 0.002 | 0.700 | 0.005 |
| Eosinophils per microliter | 0.238 | 0.671 | 0.247 |
| Platelet count per microliter | 0.306 | 0.578 | 0.361 |
| Aspartate Aminotransferase, IU/L | 0.790 | 0.914 | 0.809 |
| Alanine Aminotransferase, IU/L | 0.540 | 0.926 | 0.530 |
| Lactate dehydrogenase, IU/L | 0.011 | 0.979 | 0.018 |
| Creatine Phosphokinase, IU/L | 0.765 | 0.963 | 0.766 |
| C-reactive protein, mg/L | 0.167 | 0.361 | 0.106 |
| Creatinine, mg/dL | 0.702 | 0.734 | 0.696 |
| D-dimer, ng/mL | 0.005 | 0.637 | 0.011 |
| Ferritin, ng/mL | 0.386 | -- | 0.385 |
| Procalcitonin, ng/mL | 0.739 | 0.705 | 0.768 |
| Interleukin-6, pg/mL | 0.005 | 0.368 | 0.009 |
| Radiological findings |  |  |  |
| No radiological findings | 0.567 | 0.998 | 0.999 |
| Unilateral radiological findings | 0.998 | 0.993 | 0.998 |
| Bilateral radiological findings | 0.053 | 0.998 | 0.998 |
| Drug therapy after admission |  |  |  |
| Hydroxychloroquine | 0.999 | 0.999 | 0.999 |
| Azithromycin | 0.576 | 0.999 | 0.545 |
| Lopinavir plus ritonavir | 0.623 | 0.999 | 0.796 |
| Corticosteroids | 0.685 | 0.998 | 0.980 |
| Interferon beta | 0.999 | 1.000 | 0.999 |
| Tocilizumab | 0.001 | -- | 0.001 |

Supplemental Table S4. Effect sizes for covariates related with the admission to the intensive care unit.

|  | Overall patients  (n=113) | Patients with allergic disorders (n=24) | Other patients  (n=89) |
| --- | --- | --- | --- |
| Drugs used in cardiovascular diseases |  |  |  |
| Use of calcium channel blockers. Number of patients admitted / not admitted. | 5 / 13 | 0 / 2 | 5 / 11 |
| No use of calcium channel blockers. Number of patients admitted / not admitted. | 6 / 89 | 1 / 21 | 5 / 68 |
| Relative Risk ratio (95% C.I.), P | 4.40 (1.24-21.40), 0.005 | -- | 4.56 (1.24-16.26), 0.005 |
| Other drugs |  |  |  |
| Use of Proton pump inhibitors. Number of patients admitted / not admitted. | 9 / 45 | 1 / 6 | 8 / 39 |
| No use of Proton pump inhibitors. Number of patients admitted / not admitted. | 2 / 57 | 0 / 17 | 2 / 40 |
| Risk ratio (95% C.I.), P | 4.92 (1.06-32.61), 0.017 | -- | 3.57 (0.76-20.06), 0.069 |
| Symptoms |  |  |  |
| Headache. Number of patients admitted / not admitted. | 2 / 3 | 1 / 0 | 1 / 3 |
| No Headache. Number of patients admitted / not admitted. | 9 / 99 | 0 / 23 | 9 / 76 |
| Risk ratio (95% C.I.), P | 4.80 (1.08-49.77), 0.020 | -- | 2.36 (0.11-9.41), 0.375 |
| Laboratory and clinical findings |  |  |  |
| Oxygen saturation (%). Patients admitted to ICU. Mean ± SD | 86.60 ± 7.06 | -- | 86.60 ± 7.06 |
| Oxygen saturation (%). Patients not admitted to ICU. Mean ± SD | 92.88 ± 5.88 | -- | 92.47 ± 6.50 |
| T-test (P) | 0.002 | -- | 0.010 |
| Leukocytes per microliter. Patients admitted to ICU. Mean ± SD | 15686.36 ± 29422.26 | -- | 16655.00 ± 30828.36 |
| Leukocytes per microliter. Patients not admitted to ICU. Mean ± SD | 7845.49 ± 3792.64 | -- | 7714.94 ± 3473.49 |
| T-test (P) | 0.011 | -- | 0.013 |
| D-dimer, ng/mL. Patients admitted to ICU. Mean ± SD | 20086.55 ± 53149.72 | -- | 22040.40 ± 55606.75 |
| D-dimer, ng/mL. Patients not admitted to ICU. Mean ± SD | 2876.78 ± 9515.96 | -- | 3422.75 ± 10797.79 |
| T-test (P) | 0.005 | -- | 0.011 |
| Radiological findings |  |  |  |
| Bilateral radiological findings. Number of patients admitted / not admitted. | 10 / 57 | 0 / 16 | 10 / 41 |
| No Bilateral radiological findings. Number of patients admitted / not admitted. | 1 / 45 | 1 / 7 | 0 / 38 |
| Risk ratio (95% C.I.), P | 6.87 (0.98-143.19), 0.025 | -- | -- (P=0.004) |
| Drug therapy after admission |  |  |  |
| Use of Tocilizumab. Number of patients admitted / not admitted. | 4 / 1 | -- | 4 / 1 |
| No use of Tocilizumab. Number of patients admitted / not admitted. | 7 / 101 | 1 / 94 | 6 / 78 |
| Risk ratio (95% C.I.), P | 12.34 (3.62-17.65), 0.001 | -- | 11.20 (3.18-16.45) |

Supplemental Table S5. Univariate associations with mortality.

|  | Overall patients  (n=113) | Patients with allergic disorders (n=24) | Other patients  (n=89) |
| --- | --- | --- | --- |
| Age & gender |  |  |  |
| Age | 0.003 | 0.385 | 0.001 |
| Gender | 0.654 | 0.755 | 0.582 |
| Antecedents of allergic diseases | 0.456 |  |  |
| Drug allergy | 0.443 |  |  |
| Asthma and rhinitis | 0.999 |  |  |
| Seasonal allergy | 0.842 |  |  |
| Urticaria | 0.508 |  |  |
| Comorbidity excluding asthma or allergy | 0.998 | 0.999 | 0.999 |
| Antecedents of hypertension | 0.119 | 0.222 | 0.307 |
| Antecedents of heart diseases | 0.088 | 0.162 | 0.212 |
| Antecedents of dyslipidemia | 0.482 | 0.149 | 0.893 |
| Antecedents of type II diabetes | 0.353 | 0.149 | 0.709 |
| Antecedents of renal diseases | 0.767 | -- | 0.908 |
| Antecedents of prothrombotic disorders | 0.229 | 0.162 | 0.465 |
| Antecedents of respiratory diseases | 0.802 | 0.277 | 0.818 |
| Antecedents of cerebrovascular diseases | 0.008 | 0.149 | 0.026 |
| Antecedents of cognitive impairment | 0.199 | 0.999 | 0.132 |
| Antecedents of psychiatric diseases | 0.965 | 0.149 | 0.527 |
| Antecedents of hormonal disorders | 0.387 | 1.000 | 0.359 |
| Antecedents of neurological diseases | 0.066 | 0.149 | 0.180 |
| Antecedents of digestive diseases | 0.512 | 1.000 | 0.527 |
| Antecedents of neoplastic diseases | 0.778 | -- | 0.359 |
| Antecedents of morbid obesity | 0.999 | 0.999 | 0.999 |
| Antecedents of anaemia | 0.808 | 1.000 | 0.875 |
| Drugs used in allergic diseases | 0.811 | 0.290 | 0.448 |
| Inhaled corticosteroids | 0.905 | 0.222 | 0.622 |
| Inhaled Beta-2 agonists | 0.802 | 0.290 | 0.875 |
| Inhaled Anticholinergics | 0.712 | 0.424 | 0.958 |
| Antihistamine drugs | 0.999 | 0.999 | 0.999 |
| Anti-leukotrienes agents | 1.000 | 1.000 | -- |
| Omalizumab | 1.000 | 1.000 | -- |
| Drugs used in cardiovascular diseases |  |  |  |
| Statins | 0.280 | 0.998 | 0.964 |
| Angiotensin-II-receptor antagonists | 0.448 | 0.575 | 0.619 |
| Diuretics | 0.940 | 0.999 | 0.926 |
| Platelet Antiaggregants | 0.069 | 0.071 | 0.266 |
| Beta-blockers | 0.295 | 0.723 | 0.300 |
| Calcium channel blockers | 0.229 | 0.999 | 0.180 |
| Oral anticoagulants | 0.013 | 0.998 | 0.180 |
| Angiotensin-converting enzyme inhibitors | 0.384 | 0.149 | 0.999 |
| Other vasodilators | 0.842 | 1.000 | 0.527 |
| Pain relief medications |  |  |  |
| Non-steroidal anti-inflammatory drugs | 0.047 | 0.723 | 0.052 |
| Opiates | 0.134 | 0.071 | 0.465 |
| Psychiatric medications |  |  |  |
| Benzodiazepines | 0.030 | 0.998 | 0.292 |
| Selective serotonin reuptake inhibitors | 0.004 | 0.071 | 0.022 |
| Antipsychotic agents | 0.295 | 0.149 | 0.658 |
| Other antidepressants | 0.444 | 0.999 | 0.241 |
| Drug used in neurological diseases |  |  |  |
| Anti-Parkinson Drugs | 0.317 | -- | 0.369 |
| Antiepileptic drugs | 0.028 | 1.000 | 0.022 |
| Other drugs |  |  |  |
| Proton pump inhibitors | 0.011 | 0.998 | 0.110 |
| Oral antihyperglycemic drugs | 0.353 | 0.277 | 0.619 |
| Antibiotics | 0.387 | 1.000 | 0.359 |
| Levothyroxine | 0.213 | -- | 0.263 |
| Antiandrogens | 0.712 | -- | 0.802 |
| Oral Anticholinergics | 0.444 | -- | 0.512 |
| Adrenergic alpha 1-antagonists | 0.890 | -- | 0.958 |
| Immunosuppressants | 0.204 | 1.000 | 0.138 |
| Oral corticosteroids | 0.063 | -- | 0.074 |
| Symptoms |  |  |  |
| Fever | 0.717 | 0.999 | 0.984 |
| Dry cough | 0.391 | 0.865 | 0.403 |
| Dyspnea | 0.326 | 0.290 | 0.123 |
| Overall discomfort | 0.339 | 0.723 | 0.403 |
| Asthenia | 0.134 | 1.000 | 0.116 |
| Diarrhoea | 0.330 | 0.999 | 0.619 |
| Musculoskeletal pain | 0.900 | 0.575 | 0.713 |
| Anosmia | 0.512 | 1.000 | 0.527 |
| Ageusia | 0.842 | 1.000 | 0.802 |
| Headache | 0.890 | 1.000 | 0.999 |
| Nauseas and/or vomits | 0.699 | 0.999 | 0.301 |
| Odynophagia | 0.999 | 0.999 | 0.999 |
| Exanthema | 1.000 | -- | 1.000 |
| Laboratory and clinical findings |  |  |  |
| Temperature | 0.982 | 0.142 | 0.960 |
| Oxygen saturation | 0.001 | 0.385 | 0.001 |
| Leukocytes per microliter | 0.006 | 0.278 | 0.012 |
| Lymphocytes per microliter | 0.030 | 0.070 | 0.043 |
| Eosinophils per microliter | 0.187 | 0.600 | 0.206 |
| Platelet count per microliter | 0.320 | 0.695 | 0.358 |
| Aspartate Aminotransferase, IU/L | 0.761 | 0.618 | 0.667 |
| Alanine Aminotransferase, IU/L | 0.236 | 0.348 | 0.342 |
| Lactate dehydrogenase, IU/L | 0.032 | 0.651 | 0.025 |
| Creatine Phosphokinase, IU/L | 0.014 | 0.029 | 0.095 |
| C-reactive protein, mg/L | 0.170 | 0.453 | 0.265 |
| Creatinine, mg/dL | 0.341 | 0.001 | 0.918 |
| D-dimer, ng/mL | 0.427 | 0.450 | 0.460 |
| Ferritin, ng/mL | 0.489 | 0.328 | 0.791 |
| Procalcitonin, ng/mL | 0.022 | 0.854 | 0.027 |
| Interleukin-6, pg/mL | 0.001 | 0.006 | 0.001 |
| Radiological findings |  |  |  |
| No radiological findings | 0.196 | 0.575 | 0.999 |
| Unilateral radiological findings | 0.653 | 0.999 | 0.723 |
| Bilateral radiological findings | 0.568 | 1.000 | 0.494 |
| Drug therapy after admission |  |  |  |
| Hydroxychloroquine | 0.199 | 0.277 | 0.359 |
| Azithromycin | 0.491 | 0.999 | 0.537 |
| Lopinavir plus ritonavir | 0.011 | 0.037 | 0.174 |
| Corticosteroids | 0.339 | 0.222 | 0.622 |
| Interferon beta | 0.444 | 1.000 | 0.369 |
| Tocilizumab | 0.999 | -- | 0.999 |

Supplemental Table S6. Effect sizes for covariates related with mortality.

|  | Overall patients  (n=113) | Patients with allergic disorders (n=24) | Other patients  (n=89) |
| --- | --- | --- | --- |
| Age (years). Patients who died (years ± SD) | 80.05 ± 10.55 | 67.00 ± 13.89 | 82.35 ± 8.39 |
| Age (years). Patients who survived (years ± SD) | 64.95 ± 15.81 | 58.10 ± 16.50 | 66.94 ± 15.14 |
| T-test (P) | 0.001 | 0.385 | 0.001 |
| Comorbidities |  |  |  |
| Antecedents of cerebrovascular disorders. Patients who died / survived | 6 / 7 | 1 / 1 | 5 / 6 |
| No Antecedents of cerebrovascular disorders. Patients who died / survived | 14 / 86 | 2 / 20 | 12 / 66 |
| Relative Risk ratio (95% C.I.), P | 10.50 (0.29-223.26), 0.034 | 10.50 (0.29-223.26), 0.034 | 2.96 (1.00-6.38), 0.018 |
| Drugs used in cardiovascular diseases |  |  |  |
| Use of oral anticoagulants. Patients who died / survived | 6 / 8 | 3 / 3 | 3 / 5 |
| No use of oral anticoagulants. Patients who died / survived | 14 / 85 | 0 / 18 | 14 / 67 |
| Relative Risk ratio (95% C.I.), P | 3.03 (1.14-6.37), 0.009 | -- (P = 0.002) | 2.17 (0.53-5.24), 0.168 |
| Use of Platelet Antiaggregants. Patients who died / survived | 8 / 19 | 2 / 3 | 6 / 16 |
| No use of Platelet Antiaggregants. Patients who died / survived | 12 / 74 | 1 / 18 | 11 / 56 |
| Relative Risk ratio (95% C.I.), P | 2.12 (0.85-4.92), 0.064 | 7.60 (0.58-206.53), 0.041 | 1.66 (0.59-4.20), 0.264 |
| Pain relief medications |  |  |  |
| Use of Non-steroidal anti-inflammatory drugs. Patients who died / survived | 10 / 25 | 1 / 5 | 9 / 20 |
| No use of Non-steroidal anti-inflammatory drugs. Patients who died / survived | 10 / 68 | 2 / 16 | 8 / 52 |
| Relative Risk ratio (95% C.I.), P | 2.29 (0.93-5.29), 0.043 | 1.50 (0.05-19.22), 0.727 | 2.38 (0.90-6.01), 0.048 |
| Psychiatric medications |  |  |  |
| Use of selective serotonin reuptake inhibitors. Patients who died / survived | 9 / 14 | 2 / 3 | 7 / 11 |
| No use of selective serotonin reuptake inhibitors. Patients who died / survived | 11 / 79 | 1 / 18 | 10 / 61 |
| Relative Risk ratio (95% C.I.), P | 3.20 (1.34-7.13), 0.003 | 7.60 (0.58-206.53), 0.041 | 2.76 (1.04-6.53), 0.017 |
| Drug used in neurological diseases |  |  |  |
| Use of Antiepileptic drugs. Patients who died / survived | 3 / 2 | 0 / 1 | 3 / 1 |
| No use of Antiepileptic drugs. Patients who died / survived | 17 / 91 | 3 / 20 | 14 / 71 |
| Relative Risk ratio (95% C.I.), P | 3.81 (0.99-6.51), 0.012 | -- (P = 0.005) | 4.55 (1.19-6.43), 0.004 |
| Other drugs |  |  |  |
| Use of Proton pump inhibitors. Patients who died / survived | 15 / 39 | 3 / 4 | 12 / 35 |
| No use of Proton pump inhibitors. Patients who died / survived | 5 / 54 | 0 / 17 | 5 / 37 |
| Risk ratio (95% C.I.), P | 3.28 (1.22-9.94), 0.008 | -- (P = 0.705) | 2.15 (0.77-6.66), 0.105 |
| Laboratory and clinical findings |  |  |  |
| Oxygen saturation (%). Patients who died. Mean ± SD | 86.32 ± 9.56 | 92.00 ± 0.00 | 85.65 ± 9.92 |
| Oxygen saturation (%). Patients who survived. Mean ± SD | 93.65 ± 4.16 | 94.24 ± 3.49 | 93.44 ± 4.39 |
| T-test (P) | 0.001 | 0.385 | 0.001 |
| Lymphocytes per microliter. Patients who died. Mean ± SD | 5424.50 ± 19139.55 | 1800.00 ± 691.95 | 6064.12 ± 20785.82 |
| Lymphocytes per microliter. Patients who survived. Mean ± SD | 1131.19 ± 503.09 | 1162.86 ± 524.06 | 1121.96 ± 500.21 |
| T-test (P) | 0.030 | 0.070 | 0.043 |
| Eosinophils per microliter. Patients who died. Mean ± SD | 23.16 ± 50.89 | 16.67 ± 11.55 | 24.38 ± 55.49 |
| Eosinophils per microliter. Patients who survived ICU. Mean ± SD | 48.64 ± 81.20 | 32.86 ± 51.59 | 53.31 ± 86.62 |
| T-test (P) | 0.187 | 0.600 | 0.206 |
| Interleukin-6, pg/mL. Patients who died. Mean ± SD | 410.97 ± 335.76 | 175.30 ± 64.21 | 505.12 ± 359.57 |
| Interleukin-6, pg/mL. Patients who survived ICU. Mean ± SD | 48.17 ± 74.26 | 31.65 ± 47.33 | 51.33 ± 78.39 |
| T-test (P) | 0.001 | 0.006 | 0.001 |
| Drug therapy after admission |  |  |  |
| Use of Lopinavir plus ritonavir. Patients who died / survived | 6 / 58 | 2 / 12 | 4 / 46 |
| No use of Lopinavir plus ritonavir. Patients who died / survived | 14 / 35 | 1 / 9 | 13 / 26 |
| Risk ratio (95% C.I.), P | 0.32 (0.12-0.84), 0.008 | 1.43 (0.11-39.42), 0.759 | 0.24 (0.07-0.71), 0.003 |
